# Long terms trends of multimorbidity and association with physical activity in older English population

**DOI:** 10.1186/s12966-016-0330-9

**Published:** 2016-01-19

**Authors:** Nafeesa N. Dhalwani, Gary O’Donovan, Francesco Zaccardi, Mark Hamer, Thomas Yates, Melanie Davies, Kamlesh Khunti

**Affiliations:** Diabetes Research Centre, Leicester Diabetes Centre, Leicester General Hospital, University of Leicester, Leicester, LE5 4PW UK; National Centre Sport and Exercise Medicine, Loughborough University, Loughborough, LE11 3TU UK

**Keywords:** Multimorbidity, Physical activity, Cohort, Longitudinal trend

## Abstract

**Background:**

Multimorbidity has become one of the main challenges in the recent years for patients, health care providers and the health care systems globally. However, literature describing the burden of multimorbidity in the elderly population, especially longitudinal trends is very limited. Physical activity is recommended as one of the main lifestyle changes in the prevention and management of multiple chronic diseases worldwide; however, the evidence on its association with multimorbidity remains inconclusive. Therefore, we aimed to assess the longitudinal trends of multimorbidity and the association between multimorbidity and physical activity in a nationally representative cohort of the English population aged ≥50 years between 2002 and 2013.

**Methods:**

We used data on 15,688 core participants from six waves of the English Longitudinal Study of Ageing, with complete information on physical activity. Self-reported physical activity was categorised as inactive, mild, moderate and vigorous levels of physical activity. We calculated the number of morbidities and the prevalence of multimorbidity (more than 2 chronic conditions) between 2002 and 2013 overall and by levels of self-reported physical activity. We estimated the odds ratio (OR) and 95 % confidence intervals (CI) for multimorbidity by each category of physical activity, adjusting for potential confounders.

**Results:**

There was a progressive decrease over time in the proportion of participants without any chronic conditions (33.9 % in 2002/2003 vs. 26.8 % in 2012/2013). In contrast, the prevalence of multimorbidity steadily increased over time (31.7 % in 2002/2003 vs. 43.1 % in 2012/2013). Compared to the physically inactive group, the OR for multimorbidity was 0.84 (95 % CI 0.78 to 0.91) in mild, 0.61 (95 % CI 0.56 to 0.66) in moderate and 0.45 (95 % CI 0.41 to 0.49) in the vigorous physical activity group.

**Conclusion:**

This study demonstrated an inverse dose-response association between levels of physical activity and multimorbidity, however, given the increasing prevalence of multimorbidity over time, there is a need to explore causal associations between physical activity and multimorbidity and its impact as a primary prevention strategy to prevent the occurrence of chronic conditions later in life and reduce the burden of multimorbidity.

**Electronic supplementary material:**

The online version of this article (doi:10.1186/s12966-016-0330-9) contains supplementary material, which is available to authorized users.

## Background

Multimorbidity has become one of the main challenges in the recent years for patients, health care providers and the health care systems globally. Patients with multimorbidity have more complex health care needs and are more likely to have poorer health outcomes and lower quality of life [1] thereby increasing the health care costs associated with it [2]. To address the burden of multimorbidity, it is crucial to characterise the problem. However, literature describing the burden of multimorbidity in the elderly population reports a wide range of prevalence estimates (7 to 99 %) [3–13]. Moreover, most of these studies are cross-sectional and only one study from the Netherlands far has assessed the long term trends in multimorbidity between 1985 and 2005, which is now over a decade old [9].

Physical activity (PA) is recommended as one of the main lifestyle changes in the prevention and management of multiple chronic diseases worldwide [14]; however, the association between PA and multimorbidity has not been extensively studied and the existing evidence remains inconclusive. Two cross-sectional studies from Europe found a statistically significant between multimorbidity and physical activity in men but not women [15, 16]. Contrarily, two surveys from Canada based on 16,782 participants between 18 and 69 years and 1,196 participants over 45 years, found no statistically significant association between PA and multimorbidity [11, 17]. Given the inconsistencies in multimorbidity estimates and association with PA we aimed to assess the longitudinal trends of multimorbidity between 2002 and 2013 and the association between multimorbidity and PA in a nationally representative community based cohort of English population aged ≥50 years.

## Methods

### Data source and study population

The English Longitudinal Study of Ageing (ELSA) is an ongoing panel study of a nationally representative cohort of the English population aged ≥50 years. For the first wave of ELSA (2002–2003) participants were recruited from households that were included in the Health Survey for England in 1998, 1999 or 2001 (wave 0) [18]. The total sample for the first wave consisted for 11,391 core members [18] out of which 8,780 participants were followed in 2004/05 (wave 2). To maintain the representation of people between 50 and 53 years, refreshment samples were added in 2006/7 (wave 3), 2008/9 (wave 4), and 2012/13 (wave 6), making the total numbers of core participants for wave 3-6 to be 8,810; 9,886; 9,090 and 9,169 respectively. In addition to the follow-up assessment every two years, health examinations through nurses visit take place every four years (wave 2, 4, 6). For a detailed description of the flow of participants through ELSA please refer to the cohort profile [18]. ELSA has been shown to be broadly representative of the English population in terms of the sociodemographics [18]. In order to maintain the representation of people between 50 and 53 years, refreshment samples were added at waves 3, 4 and 6. The total number of core members interviewed at wave 6 was 9,169 [19]. ELSA collects a wide range of data on the elderly population including demographic and economic data, physical and mental health, psychosocial wellbeing, physical and cognitive function and a range of blood assays. Ethical approval for all ELSA waves was obtained from NHS Research Ethics Committees under the National Research and Ethics Service (NRES) and participants gave full informed written consent to participate in the study [20].

### Physical activity

At each wave participants were asked about the frequency of vigorous, moderate and mild PA (more than once per week, once per week, one to three times per month, or hardly ever) using show cards to help classify the intensity of each activity [20]. We categorised PA into four groups based on the classification in other ELSA studies [21, 22] i.e. inactive, only mild activity at least once a week, at least moderate but no vigorous activity at least once a week and any vigorous activity at least once a week. Mild activities included laundry and home repairs; moderate activities included gardening, cleaning the car, moderate pace walking, dancing, floor or stretching exercises etc. and vigorous activities included running or jogging, swimming, cycling, aerobics or gym workouts, tennis etc [21]. Participants with missing information on PA were dropped from the cohort.

### Multimorbidity

ELSA collects self-reported information on doctor diagnosed diabetes, hypertension, stroke, myocardial infarction, congestive heart failure, angina, lung disease, asthma, arthritis, osteoporosis, cancer, hearing problems, Parkinson’s disease, Alzheimer’s disease, dementia and eye conditions including macular degeneration and glaucoma. Chronic conditions such as diabetes have been validated in ELSA using objective biomedical data collected as part of the nurse visits [22]. We defined multimorbidity as presence of two or more of these conditions at any given time during the follow-up.

### Other covariates

We also extracted data on other covariates that have been shown to be associated with the development of chronic conditions and PA. These included age (categorised in 10 year age bands), sex, ethnicity (white, non-white and missing), total non-pension net wealth in quintile as a proxy measurement of socioeconomic status, smoking status (smoker, non-smoker), frequency of alcohol consumption in the past year (not at all, occasionally, once or twice a month, once or twice a week, at least three days a week, missing) and body mass index (BMI, categorised as underweight (<18.5 kg/m^2^), normal (≥18.5 and <25 kg/m^2^), overweight (≥25 and < 30 kg/m^2^), obese (≥30 kg/m^2^) and missing). BMI information was only collected at nurse visits at wave 0, 2, 4 and 6. For waves 1, 3 and 5 if there was a BMI recording in the preceding and subsequent waves then an average of the two BMI recordings was imputed. If there was no BMI recording in the preceding and subsequent waves then BMI was considered missing.

### Statistical analysis

We described general characteristics among participants in each wave using medians, interquartile ranges and proportions. We calculated the prevalence of each chronic condition separately for each wave and the five most common combinations of chronic conditions in the multimorbid population for each wave. We calculated the number of morbidities overall and by levels of PA, standardised by age and sex taking the sampling weights in to account. We then estimated age and sex standardised prevalence of multimorbidity for each wave as the number of participants with ≥2 conditions divided by the total number of participants in each wave, taking the cross sectional weights into account and stratifying by levels of PA. These prevalence estimates were plotted graphically to visualise long term time trends. We used a generalised estimation equation (GEE) model with an exchangeable correlation structure and calculated the odds ratios (ORs) and 95 % confidence intervals (CIs) for multimorbidity by each category of PA, adjusting for age, ethnicity, total non-pension net wealth, smoking status, alcohol consumption and BMI. All analyses were conducted in STATA 12 MP [23].

### Sensitivity analyses

People with higher levels of vigorous activity each week may also have high levels of moderate and mild activity. Therefore, we created three additional models for each level of physical activity excluding the other two and recalculated the effect estimates. Also since some studies have used the cut-off point as three or more chronic conditions to define multimorbidity we repeated our main analysis changing our definition of multimorbidity from two or more chronic conditions to three or more chronic conditions. The addition of refreshment samples (age 50–53) added at waves 3, 4 and 6 may potentially result in slight underestimation of the prevalence. Thus, we restricted the study population to the core members from wave 1 and recalculated the prevalence of multimorbidity overall and by levels of PA and also recalculated the OR for multimorbidity by each category of PA.

## Results

### General characteristics of the study population

A total of 15,783 participants contributed data to ELSA (wave 1–6) out of which 50 % contributed data to at least 3 waves. Out of these, information on PA was available for 15,688 participants who were included in this study. Table 1 presents the general characteristics of the study population for each wave. The median age of participants at baseline was 64 years (interquartile range (IQR) 56–73 years) and increased to 66 years (IQR 60–75 years) at wave 6. Just over half of the participants in each wave were females and the proportion of non-white participants was under 5 % in all the waves. 17.9 % of the participants were smokers at baseline which reduced to 14.1 % at wave 6 and approximately one third of the participants were overweight. Just under half of the study population in each wave reported some kind of moderate PA at least once a week and over a quarter engaged in vigorous PA at least once a week.

**Table 1:** Characteristics of study population for each wave

	Wave 1 *N* = 11,212	Wave 2 *N* = 8,685	Wave 3 *N* = 8,806	Wave 4 *N* = 9,877	Wave 5 *N* = 9,082	Wave 6 *N* = 9,165
	n (%)^a^	n (%)^a^	n (%)^a^	n (%)^a^	n (%)^a^	n (%)^a^
Age in years						
median (IQR)	64 (56–73)	66 (58–74)	64 (57–74)	65 (58–73)	66 (60–74)	66 (60–75)
50–59	4,125 (37.5)	2,584 (31.2)	3,045 (35.2)	2,889 (35.9)	1,967 (26.9)	2,061 (35.5)
60–69	3,352 (28.5)	2,859 (31.3)	2,597 (30.4)	3,508 (30.9)	3,503 (35.8)	3,449 (31.9)
70–79	2,530 (22.6)	2,159 (23.9)	2,038 (21.5)	2,359 (20.9)	2,414 (23.5)	2,443 (20.2)
80–89	1,106 (10.0)	988 (11.9)	991 (10.9)	985 (10.4)	1,022 (11.2)	1,005 (10.4)
90+	99 (1.4)	95 (1.7)	135 (1.9)	136 (1.7)	176 (1.9)	207 (2.0)
Sex						
Male	5,102 (46.4)	3,906 (46.1)	3,939 (46.8)	4,420 (47.0)	4,050 (46.8)	4,073 (47.4)
Female	6,110 (53.6)	4,779 (53.9)	4,867 (53.2)	5,457 (53.0)	5,032 (53.1)	5,092 (52.6)
Ethnicity						
White	4,698 (39.7)	8,484 (97.1)	8,563 (96.5)	9,566 (96.0)	8,796 (95.9)	8,841 (94.4)
Non-white	139 (1.2)	197 (2.9)	240 (3.4)	303 (3.9)	282 (4.0)	322 (5.5)
Missing	6,375 (59.2)	4 (0.03)	3 (0.03)	8 (0.06)	5 (0.04)	2 (0.05)
Total non-pension net wealth (Quintile)						
Quintile 1 (poorest quintile)	2,122 (19.5)	1,553 (19.5)	1,578 (19.5)	1,716 (19.6)	1,567 (19.7)	1,520 (19.6)
Quintile 2	2,193 (19.5)	1,703 (19.7)	1,657 (19.5)	1,882 (19.7)	1,772 (19.6)	1,717 (19.6)
Quintile 3	2,209 (19.7)	1,722 (19.8)	1,714 (19.6)	1,916 (19.4)	1,786 (19.7)	1,871 (19.7)
Quintile 4	2,215 (19.7)	1,756 (19.8)	1,723 (19.5)	1,995 (20.2)	1,834 (19.6)	1,881 (19.5)
Quintile 5 (richest quintile)	2,277 (19.7)	1,832 (19.8)	1,795 (19.5)	2,071 (19.5)	1,877 (19.6)	1,927 (19.6)
Missing	196 (1.8)	119 (1.4)	339 (2.3)	297 (2.3)	246 (1.6)	249 (1.9)
Smoking status						
Smoker	1,995 (17.9)	1,350 (16.3)	1,310 (15.6)	1,357 (15.1)	1,152 (13.9)	1,101 (14.1)
Non-Smoker	9,217 (82.1)	7,335 (83.7)	7,496 (84.4)	8,520 (84.9)	7,930 (86.1)	8,064 (85.9)
Alcohol consumption in the past year						
Not at all	1,337 (12.1)	838 (9.6)	816 (9.3)	942 (9.9)	1,014 (11.6)	1,067 (11.6)
Occasionally	2,192 (19.5)	1,207 (13.9)	1,146 (13.0)	1,317 (13.6)	1,353 (15.1)	1,282 (13.9)
Once or twice a month	1,157 (10.3)	917 (10.4)	867 (9.8)	940 (9.5)	929 (9.9)	902 (9.8)
Once or twice a week	3,376 (30.0)	1,940 (22.2)	1,808 (20.5)	2,031 (20.2)	1,863 (20.6)	1,800 (19.8)
At least three days a week	3,147 (27.9)	2,696 (30.1)	2,550 (28.9)	2,999 (29.1)	2,795 (29.8)	2,727 (28.4)
Missing	3 (0.03)	1,087 (13.4)	1,619 (18.4)	1,648 (17.7)	1,128 (12.9)	1,387 (16.4)
Body Mass Index (kg/m2)						
Underweight (<18.5)	23 (0.2)	62 (0.7)	26 (0.3)	68 (0.7)	39 (0.4)	69 (0.7)
Normal (18.5–25)	823 (7.3)	1,946 (22.1)	1,185 (13.4)	2,030 (20.0)	1,455 (16.0)	1,956 (20.9)
Overweight (25.1–30)	1,341 (11.9)	3,132 (35.7)	2,088 (23.7)	3,293 (32.6)	2,491 (27.4)	3,029 (32.5)
Obese (≥30.1)	741 (6.6)	2,085 (23.8)	1,318 (15.0)	2,457 (25.1)	1,735 (19.1)	2,327 (25.7)
Missing	8,284 (73.9)	1,463 (17.7)	4,189 (47.6)	2,029 (21.4)	3,362 (37.0)	1,784 (20.0)

^a^weighted percentage

### Description of morbidities

**Fig. 1:**
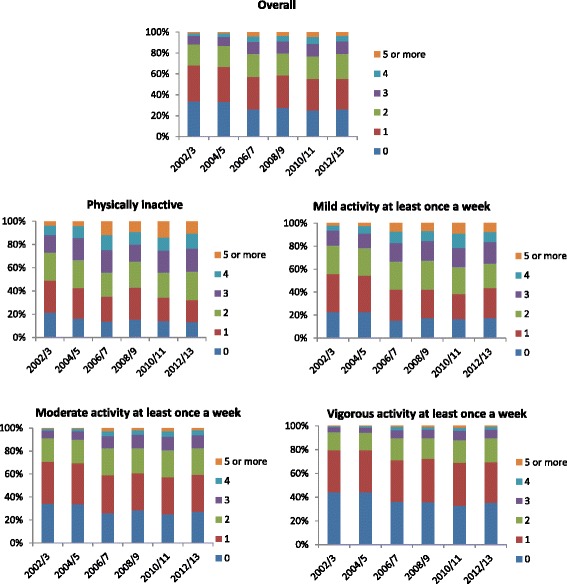
Number of morbidities by levels of physical activity, standardised by age and sex

Overall, there was a slight progressive decrease in the proportion of participants without any chronic conditions over time i.e. 33.9 % in 2002/3 compared to 26.8 % in 2012/13. In contrast, the proportion of participants with chronic morbidities increased steadily over time such that the proportion of participants with five or more morbidities was 0.9 % in 2002/3 compared to 3.9 % in 2012/13 (Fig. 1). The proportion of participants with a greater number of morbidities reduced consistently with increasing levels of PA in each wave. In 2002/3 the proportion of participants with five or more morbidities in the physically inactive group was 3.5 % compared to 0.03 % in the vigorous PA group; correspondent proportions in 2012/13 were 10.6 % in the physically inactive group compared to 0.6 % in the vigorous PA group. Generally, hypertension and arthritis were the most common chronic condition in each wave (~30 %) followed by diabetes (~9 %), asthma (~8 %) and lung disease (~6 %) (Additional file 1: Table S1). Among the participants with multimorbidity in each wave, the most common combination was hypertension and arthritis (>40 % of multimorbid participants), followed by diabetes and hypertension and asthma and arthritis in general (Additional file 2: Table S2). Although, there were slight differences in the most prevalent disease pairs within waves, the overall pattern remained very similar with most pairs comprising of hypertension, diabetes, arthritis and asthma.

### Multimorbidity over time and variations by physical activity

**Fig. 2:**
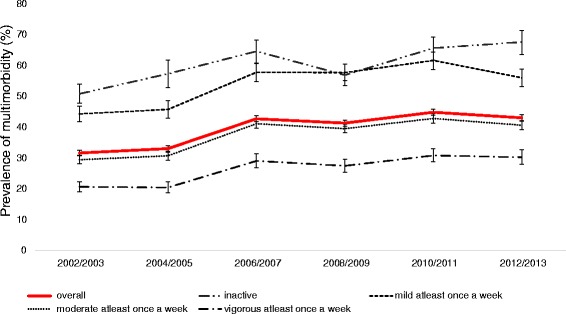
Physical activity and prevalence of multimorbidity over time

The prevalence of multimorbidity was 31.7 % (95 % CI 30.9–32.6) in 2002/3 and increased steadily over time to 43.1 % (95 % CI 42.1–44.1) in 2012/13. There was a clear gradient in the prevalence of multimorbidity by PA. Figure 2 presents the prevalence of multimorbidity over time by levels of PA. For the physically inactive group the prevalence of multimorbidity in 2002/3 was much higher at 50.9 % (95 % CI 47.9–54.0) which increased to 67.7 % (95 % CI 63.6–71.5) in 2012/13. In comparison, the prevalence of multimorbidity for the vigorous PA group was 20.7 % (95 % CI 19.1–22.4) at baseline and increased only to 30.3 % (95 % CI 28.1–32.7) in 2012/13 which was less than the multimorbidity prevalence in the physically inactive group at baseline.

**Fig. 3:**
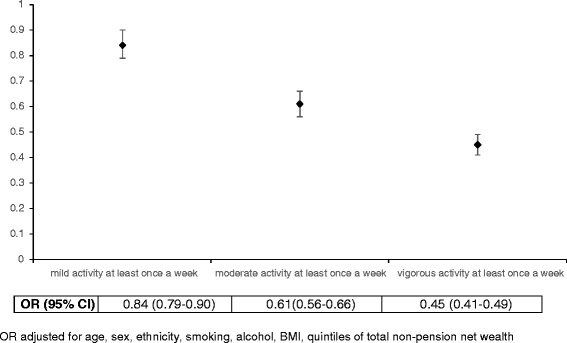
Odds of multimorbidity for each category of physical activity compared to physically inactive group

We found a dose-response association between the levels of PA and multimorbidity. Compared to the physically inactive group, the multivariable-adjusted odds of multimorbidity reduced by 16, 39 and 55 % in the mild, moderate and vigorous PA groups respectively (p-value for trend <0.001) (Fig. 3).

### Sensitivity analyses

**Fig. 4:**
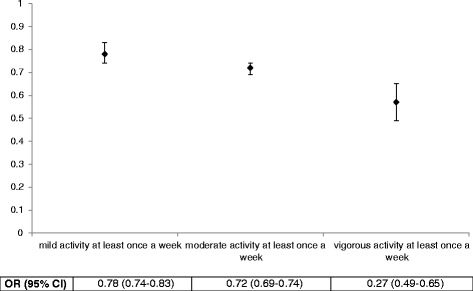
Odds of multimorbidity by each level of physical activity compared to physically inactive group, excluding the other two categories

The effect of physical activity sustained when each PA group was compared separately to physically inactive group excluding the other two PA groups. The magnitude of effect for mild PA slightly increased whilst the magnitude of effect for moderate and vigorous PA slightly decreased. After excluding any mild or moderate PA, performing vigorous PA at least once a week was found to reduce the odds of multimorbidity by 43 %(OR 0.57, 95 % CI 0.49–0.65) compared to the physically inactive group. The odds of multimorbidity reduced by 28 % (OR 0.72, 95 % CI 0.69–0.75) in the moderate PA group and 21 % in the mild PA group (OR 0.79, 95 % CI 0.75–0.83) after excluding the other two activity groups from the analysis (Fig. 4).

The prevalence of multimorbidity for each wave reduced by about 50 % when a more restrictive definition of multimorbidity (≥3 conditions) was used such that the prevalence of multimorbidity in 2002/3 was 11.7 % (95 % CI 11.1–12.3) in 2002/3 compared to 21.5 % (95 % CI 20.7–22.4) in 2012/3 (Additional file 3: Figure S1). Multimorbidity was still found to be associated with PA such that compared to the physically inactive group, the multivariable-adjusted odds of multimorbidity reduced by 17, 47 and 62 % in the mild, moderate and vigorous PA groups respectively (p-value for trend <0.001) (Additional file 4: Figure S2).

When the analysis was restricted to the core participants from the initial wave (11,298), the increase in the prevalence of multimorbidity over time was slightly more pronounced, increasing to 52.0 % (95 % CI 50.7–53.3) in 2012/13 compared to 37.6 % (95 % CI 36.7–38.4) in 2002/3 Additional file 5: Figure S3). Nevertheless, the gradient in the prevalence of multimorbidity by PA and the ORs remained the same.

## Discussion

### Principal findings

This study found that multimorbidity has increased steadily over time in the elderly English population (an increase of about 11 % between 2002 and 2013). We also found an inverse dose-response relationship between multimorbidity and levels of PA such that the odds of multimorbidity in people engaged in vigorous, moderate and mild PA at least once a week were 55, 39 and 16 % lower respectively, compared to physically inactive people.

### Strengths and limitations

Using a large population-based data source, we present longitudinal and contemporaneous estimates on multimorbidity; to our knowledge, this is the first study investigating longitudinal trends of multimorbidity in the UK and one of the very few to assess the association between multimorbidity and levels of PA. Due to the large scale and multi-purpose nature of ELSA chronic disease diagnoses are based on self-reports of physician diagnoses. However, the validity of some self-reported chronic disease diagnosis in ELSA has been demonstrated previously [24]. We further compared some of the individual chronic disease estimates from ELSA to estimates from other national data [25] and reports [26] and found good agreement with our estimates. In addition, a comparison of self-reported and physician-reported chronic conditions among multimorbid patients from the MultiCare cohort found moderate to good agreement between the two for conditions like diabetes, hypertension, stroke, asthma, COPD, osteoporosis, cancer, Parkinson’s disease etc [27]. Our estimates of multimorbidity may also have been affected by loss-to-follow up over the study time and inclusion of refreshment samples at different waves. Nevertheless, when the analyses were restricted to core participants from the initial wave our results remained unchanged. Our definition of multimorbidity only relied on the number of conditions with no account of disease severity. Nonetheless, in light of the lack of a standard definition of multimorbidity, we used the most common definition used in epidemiological studies i.e. presence of two or more chronic conditions and also restricted the definition to three or more conditions and found the results to be similar. Using a GEE model we calculated population-average estimates for the association between PA and multimorbidity; there is a potential for reverse causation and our results could arguably be interpreted as the presence of multimorbidity limiting physical activity in the population. There are also limitations with our exposure assessment. Firstly, information on PA is also self-reported in ELSA. Nevertheless, PA measures in ELSA have been validated for a subset of population using accelerometer data where self-reported PA was found to be moderately correlated with objectively assessed hours per day of moderate to vigorous PA [28]. In contrast, studies comparing self-reported PA measures to objectively measured PA found that older adults tend to over-report their PA, and vigorous activities are likely to be recalled more accurately than non-vigorous activities [29, 30]. If this were true for ELSA participants, we may have over-estimated the intensity of PA and consequently underestimated the association between PA and multimorbidity. Secondly, our classification of PA is based on the best case scenario taking the more intense PA routine for participants. However, when each activity group was analysed separately the effects still sustained with the highest protective effect in the vigorous activity group followed by moderate and mild PA. Lastly, the frequencies of mild, moderate and vigorous physical activity were only reported in categories and not true frequencies therefore we could not calculate the total minutes of PA per week to assess whether these participants met the WHO and national PA recommendations [14, 31].

### Interpretation in light of current literature

The paucity of nationally representative longitudinal studies on multimorbidity makes it difficult to compare our estimates to the current literature. To our knowledge the only study to assess longitudinal trends of multimorbidity prevalence was conducted in the Netherlands using continuous morbidity registration data and including 13,500 people between 1985 and 2005. The prevalence of multimoribidity was found to have doubled between 1985 and 2005. However, the percentage of patients with one to three conditions remained stable at about 30 % between 2003 and 2005 which is in agreement with our findings (2002/3 prevalence: 31.7 %, 2004/5 prevalence: 33.1 %) [9].

A study based on 12,611 people aged ≥65 years from the Canadian National Population Health Survey (CNPHS) found that frequent PA (defined as moderate PA lasting at least 15 min, performed ≥12 times a month) reduced the odds of the presence of chronic conditions (13 conditions included) by 9 % (OR 0.91, 95 % CI 0.89–0.93) [32]. Similarly, we found that compared to physically inactive group the odds of multimorbidity was reduced by 39 % in the moderate PA group. The higher effect estimate in our study compared to the CNPHS results can be attributed to the different definition of PA exposure. Furthermore, the CNPHS assessed presence of any chronic disease as their outcome which is different than our outcome. In contrast, a study using survey data from 1007 peopled aged 65–94 years from Germany found a 27 % reduction in the odds of multimoribidity for every 1 SD increase in PASE scores in males but no significant association between multimoribidity and PASE score in females. No gender interaction was found in our study (p-value for interaction = 0.546). Two surveys from Canada found consistently negative results contradictory to findings from our study [11, 17]. Direct comparisons to these studies are difficult due to the differences in study population, measures of PA and definitions of multimorbidity. Nevertheless our study supports a clear dose-response relationship between levels of PA and multimorbidity in geriatric English population. This association seems biologically plausible as routine physical activity has been shown to reduce abdominal adiposity and systemic inflammation; enhance lipid profiles, endothelial and cardiac function; improve glucose homeostasis, insulin sensitivity and coronary blood flow [33]. All of these factors may directly or indirectly explain the inverse association between physical activity and multimorbidity.

## Conclusion and Implications

The prevalence of multimorbidity in older adults is steadily increasing over time. The current models of care globally are based on the management of individual chronic conditions. However, given the increase in multimoribidity over the past 10 years and the complex needs of these patients clinical guidelines need to address the challenges in management of multimoribidity and formulate best practices to guide clinical decision making for multimorbid patients. The World Health Organisation recommends that older adults should do at least 150 min of moderate-intensity PA during the week or at least 75 min of vigorous-intensity PA during the week or an equivalent combination of both [14], although, a small proportion of people comply with these recommendations. This study demonstrated an inverse dose-response association between levels of physical activity and multimorbidity; however, there is a need to explore causal associations between physical activity and multimorbidity and its impact as a primary prevention strategy to prevent the occurrence of chronic conditions later in life and reduce the burden of multimorbidity.

## Additional files

Additional file 1: Table S1. Prevalence of all morbid conditions by wave (DOCX 18 kb) Additional file 2: Table S2. Five most prevalent combinations of morbidities for each wave (DOCX 16 kb) Additional file 3: Figure S1. Physical activity and age-sex standardised prevalence of multimorbidity over time using a restricted definition of multimorbidity (DOCX 16 kb) Additional file 4: Figure S2. Odds of multimorbidity for each category of physical activity compared to physically inactive group, using a restricted definition of multimorbidity (DOCX 19 kb) Additional file 5: Figure S3. Physical activity and age-sex standardised prevalence of multimorbidity over time in the restricted sample (DOCX 16 kb)
