# Supplementary material for: Long terms trends of multimorbidity and association with physical activity in older English population
Source: Int J Behav Nutr Phys Act. 2016 Jan 19;13:8. doi: 10.1186/s12966-016-0330-9 (PMC4717631; doi:10.1186/s12966-016-0330-9)
Supplement: Additional file 1: Table S1. — Prevalence of all morbid conditions by wave (DOCX 18 kb) [file 12966_2016_330_MOESM1_ESM.docx]

Supplementary Table 1 - Prevalence of all morbid conditions by wave

|  | **Wave 1 N=11,212** | **Wave 2 N=8,685** | **Wave 3 N=8,806** | **Wave 4 N=9,877** | **Wave 5 N=9,082** | **Wave 6 N=9,165** |
| --- | --- | --- | --- | --- | --- | --- |
|  | **n(%)*** | **n(%)*** | **n(%)*** | **n(%)*** | **n(%)*** | **n(%)*** |
| Diabetes | 829 (7.4) | 745 (8.5) | 842 (9.6) | 979 (9.9) | 1,057 (11.8) | 1,099 (11.4) |
| Hypertension | 4,257 (38) | 2,664 (30.7) | 3,695 (41.9) | 3,852 (38.6) | 3,775 (41.4) | 3,619 (37.4) |
| Stroke | 480 (4.3) | 282 (3.3) | 441 (5.0) | 451 (4.6) | 449 (4.9) | 455 (4.4) |
| Myocardial Infarction | 675 (5.9) | 325 (3.8) | 521 (5.9) | 586 (5.9) | 540 (5.9) | 512 (5.0) |
| Congestive Heart Failure | 24 (0.2) | 36 (0.4) | 57 (0.6) | 44 (0.4) | 49 (0.5) | 53 (0.5) |
| Angina | 671 (5.9) | 570 (6.6) | 768 (8.7) | 775 (7.8) | 848 (9.4) | 587 (5.8) |
| Lung Disease | 738 (6.5) | 459 (5.3) | 621 (7.0) | 497 (5.1) | 481 (5.4) | 468 (4.7) |
| Asthma | 993 (8.9) | 908 (10.4) | 1,006 (11.4) | 1,125 (11.3) | 1,041 (11.4) | 1,007 (10.9) |
| Arthritis | 3,659 (31.9) | 3,150 (36.3) | 3,214 (36.5) | 3,521 (34.6) | 3,489 (37.4) | 3,554 (35.0) |
| Osteoporosis | 542 (4.7) | 550 (6.3) | 580 (6.6) | 694 (6.8) | 747 (8.0) | 759 (7.4) |
| Cancer | 299 (2.6) | 378 (3.2) | 430 (4.9) | 519 (5.0) | 572 (6.0) | 540 (5.5) |
| Hearing Disorders | 563 (5.0) | 437 (5.0) | 437 (4.9) | 472 (4.7) | 466 (5.3) | 477 (4.7) |
| Parkinson’s Disease | 51 (0.4) | 17 (0.2) | 63 (0.7) | 69 (0.6) | 64 (0.6) | 63 (0.5) |
| Alzheimer’s Disease | 8 (0.07) | 8 (0.09) | 30 (0.3) | 35 (0.2) | 48 (0.4) | 52 (0.2) |
| Dementia | 43 (0.4) | 47 (0.5) | 98 (1.1) | 119 (1.0) | 140 (1.3) | 136 (1.1) |
| Eye conditions** | 650 (5.8) | 210 (2.5) | 617 (7.0) | 724 (7.1) | 786 (8.5) | 809 (7.8) |

*weighted proportions, **including macular degeneration and glaucoma
