# Supplementary material for: Long terms trends of multimorbidity and association with physical activity in older English population
Source: Int J Behav Nutr Phys Act. 2016 Jan 19;13:8. doi: 10.1186/s12966-016-0330-9 (PMC4717631; doi:10.1186/s12966-016-0330-9)
Supplement: Additional file 2: Table S2. — Five most prevalent combinations of morbidities for each wave (DOCX 16 kb) [file 12966_2016_330_MOESM2_ESM.docx]

Supplementary Table 2 - Five most prevalence combinations of morbidities for each wave

| **Wave 1** | | **Wave 2** | | **Wave 3** | | **Wave 4** | | **Wave 5** | | **Wave 6** | |
| --- | --- | --- | --- | --- | --- | --- | --- | --- | --- | --- | --- |
| **combinations** | **%** | **combinations** | **%** | **combinations** | **%** | **combinations** | **%** | **combinations** | **%** | **combinations** | **%** |
| HTN + Arthritis | 44.8 | HTN + Arthritis | 40.3 | HTN + Arthritis | 42.9 | HTN + Arthritis | 40.8 | HTN + Arthritis | 42.6 | HTN + Arthritis | 42.0 |
| HTN + Diabetes | 14.6 | Asthma + Arthritis | 14.9 | HTN + Diabetes | 15.3 | HTN + Diabetes | 16.0 | HTN + Diabetes | 17.4 | HTN + Diabetes | 18.1 |
| Asthma + Arthritis | 11.7 | HTN + Diabetes | 13.4 | HTN + Angina | 13.3 | Asthma + Arthritis | 11.9 | HTN + Angina | 13.5 | Diabetes + Arthritis | 13.0 |
| HTN + Asthma | 11.4 | Diabetes + Arthritis | 11.4 | HTN + Asthma | 12.2 | HTN + Angina | 11.9 | Diabetes + arthritis | 12.4 | Asthma + Arthritis | 11.9 |
| HTN + Angina | 10.9 | HTN + Asthma | 10.7 | Asthma + Arthritis | 12.1 | HTN + Asthma | 11.4 | Asthma + arthritis | 11.7 | Arthritis + Osteoporosis | 11.3 |
