# Supplementary material for: Long terms trends of multimorbidity and association with physical activity in older English population
Source: Int J Behav Nutr Phys Act. 2016 Jan 19;13:8. doi: 10.1186/s12966-016-0330-9 (PMC4717631; doi:10.1186/s12966-016-0330-9)
Supplement: Additional file 4: Figure S2. — Odds of multimorbidity for each category of physical activity compared to physically inactive group, using a restricted definition of multimorbidity (DOCX 19 kb) [file 12966_2016_330_MOESM4_ESM.docx]

Supplementary Figure 2 – Odds of multimorbidity for each category of physical activity compared to physically inactive group, using a restricted definition of multimorbidity

| OR (95% CI) | 0.83 (0.77-0.89) | 0.53 (0.49-0.57) | 0.38 (0.35-0.42) |
| --- | --- | --- | --- |

OR adjusted for age, sex, ethnicity, smoking, alcohol, BMI, quintiles of total wealth
