# Supplementary material for: Long terms trends of multimorbidity and association with physical activity in older English population
Source: Int J Behav Nutr Phys Act. 2016 Jan 19;13:8. doi: 10.1186/s12966-016-0330-9 (PMC4717631; doi:10.1186/s12966-016-0330-9)
Supplement: Additional file 5: Figure S3. — Physical activity and age-sex standardised prevalence of multimorbidity over time in the restricted sample (DOCX 16 kb) [file 12966_2016_330_MOESM5_ESM.docx]

Supplementary Figure 3 - Physical activity and age-sex standardised prevalence of multimorbidity over time in the restricted sample
